# Joint Modeling of Birth Outcomes Using a Copula Distributional Regression Approach

**DOI:** 10.1002/hec.70067

**Published:** 2025-12-01

**Authors:** Giampiero Marra, Rosalba Radice

**Affiliations:** ^1^ Department of Statistical Science University College London London UK; ^2^ Bayes Business School City St George's, University of London London UK

**Keywords:** birth outcomes, copula regression, joint modeling, low birth weight, maternal risk factors, preterm birth, spatial effects

## Abstract

Low birth weight and preterm birth are key indicators of neonatal health, influencing both immediate and long‐term infant outcomes. While low birth weight may reflect fetal growth restrictions, preterm birth captures disruptions in gestational development. Ignoring the potential interdependence between these variables may lead to an incomplete understanding of their shared determinants and underlying dynamics. To address this, a copula distributional regression framework is adopted to jointly model both indicators as flexible functions of maternal characteristics and geographic effects. Applied to female birth data from North Carolina, the methodology identifies shared factors of low birth weight and preterm birth, and reveals how maternal health, socioeconomic conditions and geographic disparities shape neonatal risk. The joint modeling approach provides a more nuanced understanding of these birth metrics, offering insights that can inform targeted interventions, prenatal care strategies and public health planning.

## 1 Introduction

Preterm birth and low birth weight are important indicators of maternal and infant health, widely used to assess the quality of care, the healthcare system performance and long‐term child outcomes. Preterm birth, defined as birth before 37 weeks of gestation, affected an estimated 13.4 million newborns globally in 2020, with prevalence rates varying between 4% and 16% across countries (World Health Organization 2023). Similarly, low birth weight, defined as a birth weight below 2.5 kg, was reported in approximately 19.8 million cases worldwide in 2020, accounting for 14.7% of all births (UNICEF 2023). These metrics are often associated with neonatal mortality and long‐term health issues. Preterm birth complications are the leading cause of death among children under five, responsible for approximately 900,000 fatalities in 2019 (World Health Organization 2023). Furthermore, preterm birth and low birth weight are linked to neurodevelopmental impairments, as well as an increased risk of cardiovascular disease and metabolic disorders in adulthood (Raju et al. 2017; Clayton et al. 2025). Moreover, low birth weight infants tend to be at higher risk of stunted growth, reduced cognitive ability and chronic conditions such as obesity and diabetes later in life (UNICEF 2023; Jornayvaz et al. 2016).

Socioeconomic and racial disparities significantly influence the incidence of preterm birth and low birth weight. Research highlights that maternal education, income, housing stability, financial stress and access to healthcare services play essential roles in pregnancy outcomes (Kramer and Hogue 2009; Kaiser Family Foundation 2024). Even in high‐income countries with universal healthcare, disparities persist, suggesting that non‐medical factors such as structural inequalities and social determinants of health contribute to the problem. In the United States, for example, the preterm birth rate in 2022 was 14.6% among Black women, compared with 9.4% among White and 10.1% among Hispanic women (Centers for Disease Control and Prevention 2023).

Given the complex interplay between maternal characteristics, socioeconomic conditions and perinatal indices, analyzing preterm birth and low birth weight in isolation risks overlooking important dependencies. To address this, the current study employs a copula distributional regression framework (Marra and Radice 2025a), which enables the joint modeling of both birth metrics while accommodating flexible, potentially nonlinear relationships between predictors and responses. This approach supports a more comprehensive understanding of infant health and allows for the estimation of response probabilities under varying maternal profiles. The modeling framework is implemented in the R package GJRM (Marra and Radice 2025b), which provides tools for fitting the adopted copula model and generating intuitive numerical and visual summaries.

The empirical analysis utilizes data from female infant births in North Carolina, investigating the joint distribution of preterm birth and low birth weight while accounting for maternal attributes and spatial effects. The modeling framework captures complex dependencies between these outcomes, providing insights into high‐risk groups and highlighting regions where structural inequalities, environmental exposures or healthcare access may elevate risks. Understanding these patterns can support the design of targeted public health interventions to reduce disparities and support maternal and infant well‐being.

The remainder of the paper is structured as follows. Section 2 describes the dataset and the variables used in the case study. Section 3 introduces the copula distributional regression framework, outlines the model specification and discusses the implementation in R. Section 4 presents the empirical results and discusses their practical implications for public health policy and clinical practice. Finally, Section 5 summarizes the main findings and considers directions for future research.

## 2 The Data

The dataset consists of a representative sample of 20,000 female infant births in North Carolina in 2008, compiled by the North Carolina State Center for Health Statistics (https://schs.dph.ncdhhs.gov/), and includes detailed information on maternal characteristics and key infant birth outcomes, which are described in Table 1.

**TABLE 1:** Description of variables.

Variable	Description
Weight	Infant's birth weight (kg).
Gestation	Completed weeks of gestation.
lbw	1 if birth weight < 2.5 kg, 0 otherwise.
ptb	1 if gestation period < 37 weeks, 0 otherwise.
Ethnicity	Categorical variable with four categories: White, hispanic, Black, other.
Educ	Mother's education level: Primary, Secondary, tertiary.
Firstbirth	1 if it is the mother's first birth, 0 otherwise.
Marital	1 if married, 0 otherwise.
Smoke	1 if the mother is a smoker, 0 otherwise.
age	Age of the mother (years).
County	Numeric code identifying the North Carolina county where the birth occurred (1–100).

**FIGURE 1:**
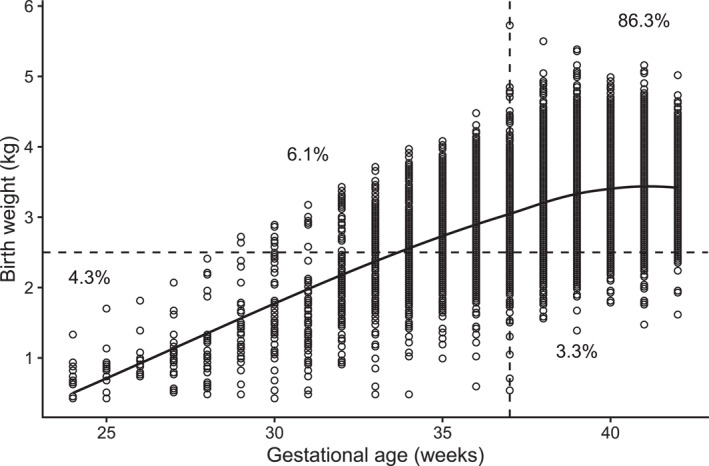
Scatterplot of birth weight (kg) versus gestational age (weeks) for the study population. The vertical line at 37 weeks and the horizontal line at 2.5 kg indicate the clinical thresholds for preterm birth and low birth weight, respectively. Quadrants corresponding to the four possible binary outcomes are labeled with the percentage of infants falling in each category. A LOESS curve is shown to highlight the association between the variables.

The focus is on the relationship between low birth weight and preterm birth, and how these outcomes are influenced by maternal and geographic characteristics. Figure 1 presents a scatterplot of birth weight versus gestational age. The relationship is positive up to roughly 37 weeks, after which it plateaus, indicating that additional gestational time contributes little to further growth. In other words, the observed association between the two variables is largely driven by values below the clinical thresholds. Overall, 86.3% of infants lie above both cut points, while 13.7% fall below one or both. Moreover, approximately 4.3% of infants are close to the cutoffs (within ±1 week of 37 weeks or ±0.2 kg of 2.5 kg). Given the quantitative nature of birth weight and gestational age, a copula regression model with continuous margins was initially explored. Several two‐ and three‐parameter distributions were considered for each outcome (including log‐normal, Gumbel, Weibull, inverse Gaussian, Dagum and Singh‐Maddala), but none provided an adequate fit to the observed responses. Combined with the fact that relatively few observations lie near the clinical thresholds, a binary modeling framework was ultimately deemed reasonable and interpretable, while also aligning with conventional clinical definitions. The work of Neelon et al. (2014) is the most directly comparable to the present study. Other investigations examining similar outcomes or methods, providing complementary insights, include Muche et al. (2024); Lean et al. (2021); Ngwenya et al. (2021); Mendoza et al. (2021); Lagendijk et al. (2020); Griffin et al. (2018); Karlsen et al. (2016); Feng (2015).

A joint regression analysis of lbw and ptb makes it possible to assess how maternal characteristics and geographic variation shape both responses simultaneously, capturing their potential synergy and providing a more realistic picture of high‐risk groups.

## 3 Modeling Framework

Let $Y_{1}$ and $Y_{2}$ denote binary outcomes for low birth weight and preterm birth, respectively, where $Y_{j}∼\text{Bernoulli}\left(\mu_{j}\right)$, for j=1,2, and $\mu_{j}$ is related to covariate effects via a link function. The joint probability distribution of $Y_{1}$ and $Y_{2}$ can be expressed as $P\left(Y_{1}=1,Y_{2}=1\right)=C\left(F_{1}\left(1;\mu_{1}\right),F_{2}\left(1;\mu_{2}\right);\theta \right)$, where $F_{j}\left(1;\mu_{j}\right)$ is the marginal cumulative distribution function (CDF) of $Y_{j}$ evaluated at one, and C(⋅,⋅;θ) is a copula function capturing the association between the responses, with a dependence parameter θ that can also be modeled as a function of regressors.

The main advantage of the copula approach is that it enables the construction of multivariate distributions from arbitrary marginal CDFs and a specified dependence function, while allowing separate modeling of the marginal and dependence structures if desired. Identifiability of the copula function can be problematic when the outcomes are not continuous, as is the case here. Nevertheless, as noted by Trivedi and Zimmer (2007) and Yang et al. (2020), this issue rarely arises in regression models with one or more continuous covariates, which effectively transform $F_{1}\left(1;\mu_{1}\right)$ and $F_{2}\left(1;\mu_{2}\right)$ from discrete points into continuous intervals, introducing variation to identify the copula across their ranges.

Further details on this type of modeling can be found in Marra and Radice (2025a).

### 3.1 Model Building and Selection

Marginal models were specified first, with their dependence examined subsequently. All the regressors listed in Table 1 were considered in the formulation of the copula distributional regression model. Covariate effects were specified through flexible predictors, incorporating smooth functions of age, to capture potential nonlinear relationships, and Markov random field (MRF) smooth terms for the county variable. The MRF approach allows spatially adjacent counties to have similar estimated impacts while preserving differences where supported by the data (see Section 1.1, Marra and Radice 2025a, for details). Categorical variables were represented using standard dummy (indicator) coding, with separate parameters estimated for each category level. Interaction terms were not included to maintain parsimony and interpretability, although the modeling framework can accommodate them if warranted.

For lbw and ptb, Bernoulli distributions with probit, logit and cloglog link functions were considered, with model selection guided by the Akaike and Bayesian information criteria. The probit link was slightly preferred.

The dependence between the marginals was modeled using the copula functions listed in Marra and Radice (Section 1.1, 2025a), including the Gaussian, Clayton, Joe, Gumbel, Frank, Farlie‐Gumbel‐Morgenstern and Plackett. A Gaussian copula was initially fitted, with θ expressed as a function of covariate effects, analogous to the marginal regressions, and then progressively simplified, as most covariates exhibited negligible influence. Since the estimated correlation was positive across all observations, the exploration of alternative dependence structures was limited to copulae supporting only positive association. With minimal differences indicated by information criteria, the Gaussian copula was ultimately adopted, which, together with probit margins, yields the well‐known bivariate probit model.

### 3.2 Model Equations

The selected equations for the outcome probabilities $\mu_{j}$ and the dependence parameter θ are

$$\Phi^{-1}\left(\mu_{j}\right)=\beta_{0\mu_{j}}+\beta_{1\mu_{j}}\;{ethnicity}_{\text{Hispanic}}+\beta_{2\mu_{j}}\;{ethnicity}_{\text{Black}}+\beta_{3\mu_{j}}\;{ethnicity}_{\text{Other}}+\beta_{4\mu_{j}}\;{educ}_{\text{Secondary}}+\beta_{5\mu_{j}}\;{educ}_{\text{Tertiary}}+\beta_{6\mu_{j}}\;{firstbirth}_{\text{Yes}}+\beta_{7\mu_{j}}\;{marital}_{\text{Married}}+\beta_{8\mu_{j}}\;{smoke}_{\text{Yes}}+s_{1j}\left(age\right)+s_{2j}\left(county\right),\;j=1,2,$$

and

$$\text{atanh}\left(\theta \right)=\beta_{0\theta }+\beta_{1\theta }\;{educ}_{\text{Secondary}}+\beta_{2\theta }\;{educ}_{\text{Tertiary}}.$$

Here, $\Phi^{-1}\left(\cdot \right)$ denotes the inverse of the standard normal CDF, which ensures that $\mu_{j}$ remains within the range (0,1), $\text{atanh}\left(\theta \right)=1/2\;\ln\left(\frac{1+\theta }{1-\theta }\right)$ is the inverse hyperbolic tangent, which maps θ from (−1,1) to the real line, maternal age effects are modeled flexibly using penalized thin‐plate regression splines and geographic effects are captured using MRF smooth terms.

### 3.3 Implementation in R

Model estimation and inference were conducted using the R package GJRM (Marra and Radice 2025b), which provides comprehensive tools for fitting copula‐based models and generating interpretable numerical and graphical summaries. The model was implemented using



library(GJRM)
eqmu1 <‐ lbw ∼ ethnicity + educ + firstbirth         + marital + smoke + s(age) +         s(county, bs = "mrf", xt = xt)
eqmu2 <‐ ptb ∼ ethnicity + educ + firstbirth         + marital + smoke + s(age) +         s(county, bs = "mrf", xt = xt)
eqthe <‐    ∼ educ
out <‐ gjrm(list(eqmu1, eqmu2, eqthe), margins = c("probit", "probit"), copula = "N", data = infants, model = "B")



where model = “B” specifies a bivariate model, s() is employed to include smooth terms for continuous or spatial covariates and the remaining quantities and options have the obvious interpretation. For spatial effects, s(, bs = “mrf”, xt = xt) implements an MRF smooth, with xt providing the neighborhood structure of adjacent units. Post‐estimation functions such as conv.check(), copula.prob(), summary(), AIC() and plot() can be employed to check for convergence and extract numerical and visual summaries.

Jointly analyzing the outcomes of interest enables a probabilistic characterization beyond what separate univariate regressions provide. While covariate effects in the marginal equations of a bivariate model are usually very similar to those from univariate models, the simultaneous approach allows for a detailed assessment of how joint and conditional probabilities vary with individual characteristics, for example, estimating the likelihood of low birth weight given preterm birth, normal birth weight given preterm birth or preterm birth given full term.

## 4 Case Study Results

The following sections present the results of the empirical analysis, focusing on the estimated dependence between low birth weight and preterm birth, the impact of key regressors, model‐based statistics and their practical implications.

### 4.1 Dependence

The overall Gaussian copula parameter $\hat{\theta }=0.74$, with 95% interval (0.70,0.77), represents the correlation between the underlying continuous latent variables that generate the observed binary outcomes of low birth weight and preterm birth (i.e., the tetrachoric correlation). This coefficient ranges from −1 to 1, with positive values indicating that a higher propensity for one outcome is associated with a higher propensity for the other. Thus, the estimate indicates a strong positive latent‐level association between the responses. This confirms that infants born preterm are highly likely to also have low birth weight, reflecting the compounded health risks faced by this population.

As shown in Table 2, tertiary‐educated mothers show a significant increase in the correlation compared to primary‐educated mothers. This does not imply that higher education increases biological risk; rather, it likely reflects differences in healthcare utilization and detection. More educated mothers often access prenatal care more frequently and intensively, which can lead to earlier identification of fetal growth restrictions or other complications. Consequently, medical interventions, such as scheduled early deliveries, may heighten the chance that preterm infants are also low birth weight. In contrast, less educated mothers may experience preterm births without such interventions, resulting in a wider range of birth weights among preterm infants. Therefore, the stronger correlation in tertiary‐educated mothers is likely driven by patterns of medical monitoring and intervention rather than a direct adverse effect of maternal education.

**TABLE 2:** Estimated coefficients for the copula dependence parameter θ, based on a Gaussian copula distributional regression model with probit margins fitted to the infant data.

Copula correlation parameter				
Parameter	Estimate	Std. error	Z value	*p*‐value
Intercept	0.670	0.129	5.194	<0.001
educSecondary	0.205	0.133	1.543	0.123
educTertiary	0.374	0.134	2.784	0.005

### 4.2 Impacts of Regressors on $\mu_{1}$ and $\mu_{2}$

Tables 3 and 4 highlight several determinants influencing the likelihood of lbw and ptb.

**TABLE 3:** Estimated coefficients on the scale of the predictor of $\mu_{1}$, based on a Gaussian copula distributional regression model with probit margins fitted to the infant data.

Low birth weight				
Parameter	Estimate	Std. error	*Z* value	*p*‐value
Intercept	−1.611	0.094	−17.110	<0.001
ethnicityHispanic	−0.033	0.048	−0.693	0.488
ethnicityBlack	0.376	0.034	11.092	<0.001
ethnicityOther	0.250	0.063	3.968	<0.001
educSecondary	0.047	0.090	0.528	0.598
educTertiary	−0.042	0.093	−0.457	0.648
firstbirthYes	0.129	0.030	4.357	<0.001
maritalMarried	−0.087	0.033	−2.649	0.008
smokeYes	0.427	0.041	10.544	<0.001

**TABLE 4:** Estimated coefficients on the scale of the predictor of $\mu_{2}$, based on a Gaussian copula distributional regression model with probit margins fitted to the infant data.

Preterm birth				
Parameter	Estimate	Std. error	*Z* value	*p*‐value
Intercept	−1.253	0.079	−15.822	<0.001
ethnicityHispanic	−0.029	0.042	−0.688	0.492
ethnicityBlack	0.283	0.032	8.905	<0.001
ethnicityOther	0.119	0.060	1.980	0.048
educSecondary	−0.021	0.075	−0.284	0.777
educTertiary	−0.103	0.078	−1.325	0.185
firstbirthYes	−0.010	0.027	−0.374	0.708
maritalMarried	−0.084	0.030	−2.765	0.006
smokeYes	0.162	0.040	4.073	<0.001

Infants born to mothers who are Black or of Other ethnicities are more likely to have low birth weight compared to those born to White mothers, while infants of Hispanic mothers do not differ significantly from those of White ones. This pattern is consistent with epidemiological evidence from the United States, where racial disparities in birth outcomes are well‐documented and often attributed to a combination of socioeconomic factors, differential access to healthcare and stress associated with structural inequalities. Parity, measured here as whether the infant is the mother's first birth, also plays a significant role. First‐born infants have a higher probability of low birth weight, which is biologically plausible: the maternal body undergoes physiological adaptations in subsequent pregnancies that can enhance fetal growth, and first‐time pregnancies tend to have slightly lower birth weight due to such natural physiological differences. Marital status is another contributing factor; infants of married mothers have a slightly lower chance of being low birth weight, likely reflecting the protective effects of greater social and economic support, which can influence maternal nutrition, stress levels and access to healthcare. Maternal smoking substantially increases the probability of low birth weight, which aligns with well‐established evidence that smoking during pregnancy restricts fetal growth. Maternal education, however, does not show a significant effect on birth weight: while higher education is often linked to greater health knowledge, income and access to care, birth weight is influenced by many biological factors, therefore differences in maternal education do not always translate into variation in infant birth weight.

Regarding ptb, maternal race, marital status and smoking again emerge as significant predictors. Black and Other ethnicities are associated with a higher probability of preterm birth, while being married is associated with a lower probability and maternal smoking increases the risk. These findings are consistent with prior literature. Unlike low birth weight, however, first birth does not significantly influence preterm birth. The lack of association with first birth aligns with biological knowledge: while first‐time pregnancies are more likely to produce smaller infants, the timing of gestation may be less sensitive to parity. The non‐significant effect of maternal education may again reflect that gestational duration is influenced more by biological and behavioral factors than by education itself, or that any potential effects of education are mediated indirectly through health behaviors, access to care and other socio‐economic factors rather than directly impacting the timing of birth.

**FIGURE 2:**
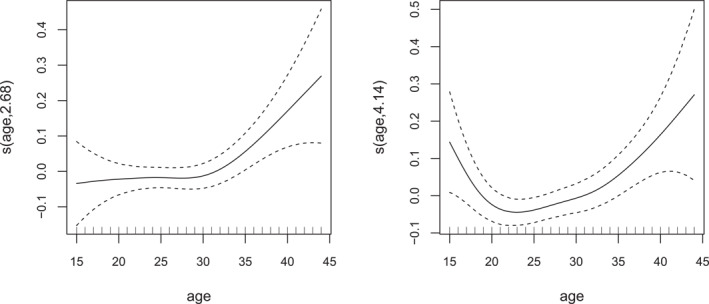
Estimated smooth effects of age on the predictor scales of $\mu_{1}$ (left) and $\mu_{2}$ (right), based on a Gaussian copula distributional regression model with probit margins fitted to the infant data. The *y*‐axes show the contributions of the smooths of age to lbw and ptb, with 95% intervals. Numbers in parentheses in the smooth labels (e.g., s(age, 2.68)) indicate the estimated degrees of freedom, reflecting the smooth's flexibility: higher values correspond to more flexible fits.

The plots in Figure 2 illustrate the effects of maternal age on the birth outcomes. The relationship between age and low birth weight is fairly flat up to about age 30, after which the likelihood of low birth weight begins to increase. This pattern for older mothers may reflect age‐related physiological changes, a higher prevalence of pregnancy complications or pre‐existing health conditions that can affect fetal development. For preterm birth, the relationship follows a U‐shaped trend, with higher likelihoods observed for both younger and older mothers relative to those in the middle of the reproductive years. In our sample, about 12% of mothers are adolescents (under 20 years) and roughly 11% are 35 or older. This suggests that shorter gestation among adolescents may reflect biological immaturity and socioeconomic factors, whereas older mothers may experience complications such as gestational hypertension and reduced placental efficiency.

**FIGURE 3:**
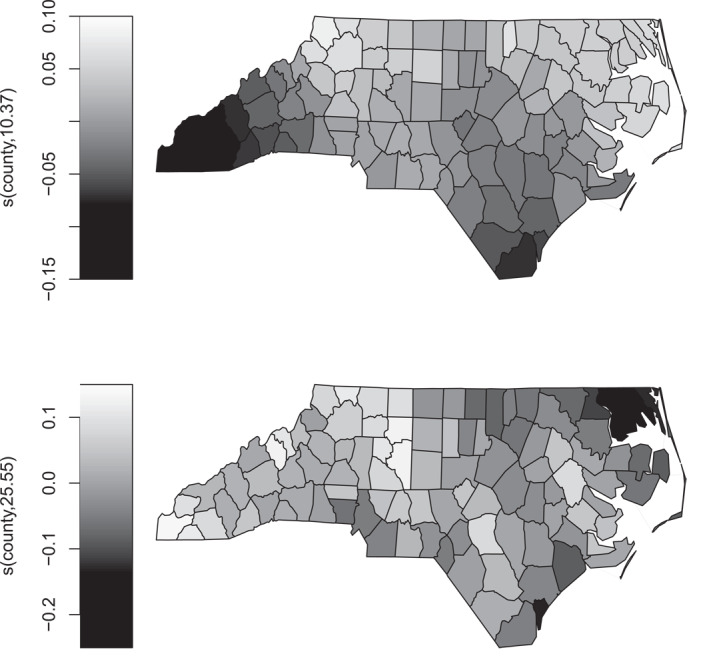
Estimated effects of county on the predictor scales of $\mu_{1}$ (top) and $\mu_{2}$ (bottom), based on a Gaussian copula distributional regression model with probit margins fitted to the infant data. Numbers in parentheses in the smooth labels indicate the estimated degrees of freedom, reflecting the smooth's flexibility: higher values correspond to more flexible fits.

Figure 3 illustrates the geographic effects, showing that the probability of low birth weight is lower in western North Carolina, which may be related to regional differences in maternal health, prenatal care utilization and environmental factors. Conversely, the likelihood of preterm birth appears to be lower in some northeastern areas, potentially reflecting variations in healthcare infrastructure, maternal education initiatives and other local factors.

As expected, fitting two separate univariate models for the birth outcomes, using the same specifications employed for the marginal equations of the copula model, produced very similar results for both the parametric coefficients and smooth effects (results not reported).

### 4.3 Model‐Based Statistics

Marginal probabilities of a female infant being preterm or having low birth weight can be estimated based on maternal characteristics. For example, for a representative mother (26 years old, White, with tertiary education, residing in Mecklenburg, married, non‐smoker and not experiencing their first birth), the results indicate a 3.97% probability of preterm birth, with 95% interval (3.25,4.79), and a 6.34% (5.37,7.52) probability of low birth weight. These estimates are lower than global averages but consistent with expectations for high‐income settings such as the United States.

**FIGURE 4:**
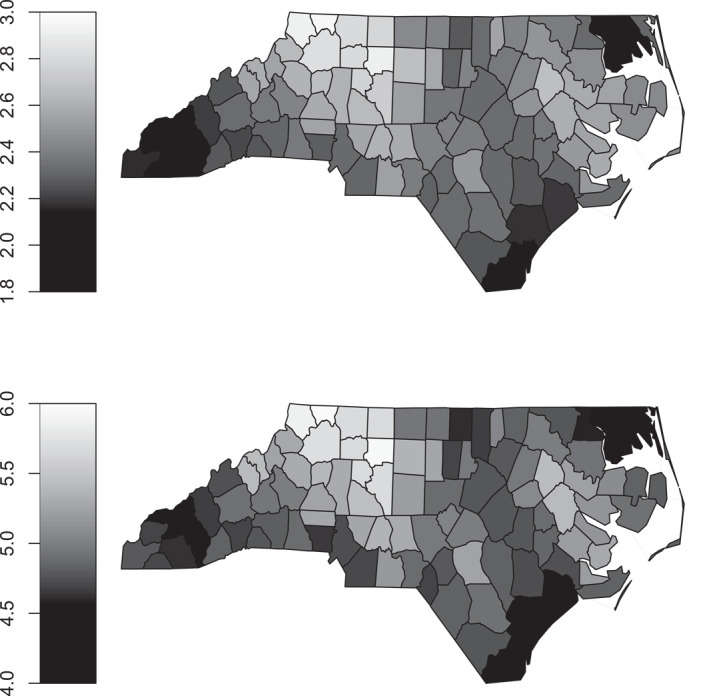
Estimated probabilities (expressed as percentages) of P(lbw=1,ptb=1) for a female infant born to a representative individual (26‐year‐old White mother with tertiary education, married and not experiencing their first birth) by county. Estimates are obtained from a Gaussian copula distributional regression model with probit margins fitted to the infant data. The top panel displays the results for a representative non‐smoking subject, while the bottom panel presents corresponding estimates for the same mother under the scenario of maternal smoking.

**FIGURE 5:**
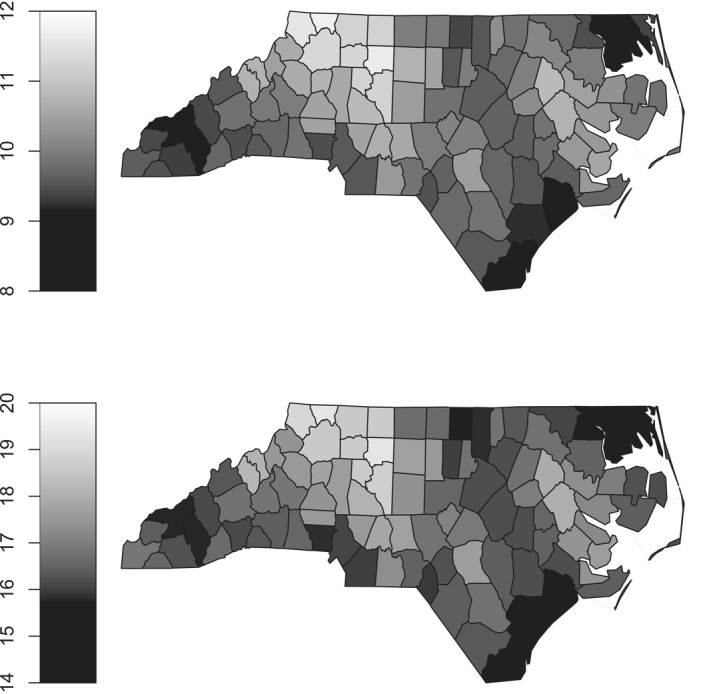
Estimated probabilities (expressed as percentages) of P(lbw=1,ptb=1) for a female infant born to a high‐risk individual (42‐year‐old Black mother with primary education, unmarried and experiencing their first birth) by county. Estimates are obtained from a Gaussian copula distributional regression model with probit margins fitted to the infant data. The top panel displays the results for a non‐smoking high‐risk subject, while the bottom panel presents corresponding estimates for the same mother under the scenario of maternal smoking.

Joint probabilities, such as P(lbw=1,ptb=1), can be straightforwardly derived from the fitted model. The top panel of Figure 4 depicts the geographic variation in this probability for the representative mother described above, highlighting counties where infants face a higher risk of being both preterm and low birth weight. For example, counties such as Ashe, Alleghany, Surry, Wilkes and Yadkin exhibit some of the highest joint probabilities, suggesting that localized factors, such as disparities in healthcare access, socioeconomic conditions and environmental exposures, may contribute to these elevated risks. The bottom panel of Figure 4 shows the corresponding joint probabilities for a female infant born to a smoking mother, revealing a clear increase in risk compared with non‐smoking mothers. This pattern is consistent with established evidence that maternal smoking is a major risk factor for adverse birth outcomes, stressing its detrimental effects across all geographic areas. For comparison, joint probabilities are also estimated for a high‐risk maternal profile (42‐year‐old Black mother with primary education, unmarried and experiencing their first birth). The estimates are reported in Figure 5 by county, with the results shown for both non‐smoking and smoking scenarios. When comparing maternal profiles, the increase in the probability is substantial for the high‐risk mother. For non‐smoking mothers, the probabilities for the representative individual range from 1.8% to 3%, while for a high‐risk mother they range from 8% to 12%. This is roughly a fourfold relative increase and an absolute increase of 6–9 percentage points. Among smoking mothers, the probabilities rise from 4% to 6% for the representative mother to 14%–20% for the high‐risk mother, corresponding to a threefold relative increase and an absolute increase of 10–14% points. These comparisons illustrate that high‐risk maternal characteristics substantially elevate both the relative and absolute risk of unfavorable birth outcomes, with some variation across counties reflecting regional differences in maternal health and healthcare access. Of course, other maternal profiles could be examined, such as those of adolescents, to explore how risks vary across different groups. Overall, the results are context‐dependent, highlighting how combinations of maternal characteristics, behaviors and geographic location interact to shape the probabilities of these birth metrics.

Conditional probabilities, such as P(lbw=1|ptb=1), provide insights into the interdependence between the birth indicators. The probability that an infant is low birth weight given birth preterm is estimated at 36.60% (31.53, 41.90). In contrast, when the infant is not preterm, the probability of low birth weight drops markedly to 1.77% (1.34, 2.29). This sharp difference highlights the strong conditional association between gestational duration and fetal growth.

The adopted modeling framework explicitly captures the joint behavior of the birth outcomes, offering a more nuanced understanding of how risks co‐occur and compound. The copula‐based approach thus provides an alternative perspective on perinatal health by emphasizing the interconnections between preterm birth and low birth weight, enabling a more integrated assessment of risk and supporting the development of targeted, multifaceted prevention strategies.

### 4.4 Practical Implications

The empirical findings offer several insights that may be relevant for maternal and infant health, particularly in contexts where understanding the joint behavior of preterm birth and low birth weight can complement existing approaches to risk assessment and planning. While the analysis is observational and based on population‐level patterns, the results highlight areas where a joint modeling framework may support further investigation and inform discussions in public health and clinical practice.

More specifically, the joint model may assist in:

- Characterizing co‐occurring risks. By quantifying how preterm birth and low birth weight tend to occur together, the framework provides probabilistic summaries that go beyond separate univariate analyses, offering a fuller picture of how maternal factors relate to both outcomes simultaneously.
- Exploring maternal profiles associated with elevated risk. The analysis illustrates how combinations of characteristics, such as smoking, marital status, ethnicity and age, are associated with joint and conditional probabilities of adverse outcomes. These patterns, while not prescriptive, may help researchers and practitioners identify profiles that warrant closer attention.
- Examining geographic variation. The spatial patterns in the joint probabilities highlight counties where the combined risk is comparatively higher. Such information may be used alongside existing local knowledge to support discussions on service provision, prenatal care accessibility and regional health inequalities.
- Complementing existing risk assessment tools. Joint modeling can provide an additional layer of information for exploring alternative scenarios or sensitivity analyses, potentially supporting decision‐making processes in settings where both outcomes are routinely monitored.

These points illustrate how a copula‐based framework can enrich the interpretation of perinatal data by integrating dependence between key birth outcomes. The approach is not intended as a replacement for established clinical or policy tools, but rather as a complementary analytic perspective that may encourage more holistic evaluations of maternal and infant health risks.

While these findings provide critical insights into maternal and infant health, it is important to recognize that they are based on population‐level estimates and a limited set of maternal characteristics. Individual pregnancies may be influenced by additional biological, environmental and socioeconomic factors that are not fully captured in this study. For example, maternal nutrition, body mass index, pre‐existing health conditions (e.g., hypertension and diabetes), prenatal care utilization, exposure to environmental pollutants, psychosocial stress and neighborhood‐level deprivation could all play important roles in shaping birth outcomes. At the same time, the bivariate model helps capture the association between preterm birth and low birth weight that is not explained by the observed covariates.

## 5 Conclusions

This study applied a copula distributional regression framework to jointly model low birth weight and preterm birth, capturing the inherent dependencies between these important perinatal outcomes. The adopted approach provides a detailed understanding of how risk factors, such as maternal smoking, ethnicity, marital status and geographic location, influence these birth outcomes. Beyond its explanatory power, this framework may serve as a decision‐support tool, enabling clinicians and public health officials to estimate joint and conditional probabilities of interest for different maternal profiles. The resulting insights can help guide targeted prenatal interventions, optimize maternal healthcare resources and inform policies aimed at reducing disparities in birth outcomes.

The strong association between preterm birth and low birth weight, as reflected by the estimated association parameter, highlights the value of considering these metrics jointly. While traditional univariate models provide insights into each outcome individually, the copula approach complements them by offering a more comprehensive perspective on risk.

The methodology presented in this article is implemented in the freely available R package GJRM, which supports model estimation, reproducibility and intuitive interpretation through numerical and visualization tools. Future research could extend this framework of analysis by incorporating further birth indicators, such as stillbirth and neonatal death. Modeling these additional outcomes could reveal severe fetal and neonatal risks, thereby informing better risk assessment, targeted prenatal care and maternal‐fetal health strategies.

## Conflicts of Interest

The authors declare no conflicts of interest.

## Data Availability

The data that support the findings of this study are available from North Carolina State Center for Health Statistics. Restrictions apply to the availability of these data, which were used under license for this study. Data are available from https://schs.dph.ncdhhs.gov/ with the permission of North Carolina State Center for Health Statistics.
